# Mechanotransduction, nuclear architecture and epigenetics in Emery Dreifuss Muscular Dystrophy: tous pour un, un pour tous

**DOI:** 10.1080/19491034.2018.1460044

**Published:** 2018-05-08

**Authors:** Andrea Bianchi, Pierluigi Giuseppe Manti, Federica Lucini, Chiara Lanzuolo

**Affiliations:** a CNR Institute of Cell Biology and Neurobiology, Istituto di Ricovero e Cura a Carattere Scientifico Fondazione Santa Lucia, Rome, Italy; b Istituto Nazionale Genetica Molecolare Romeo ed Enrica Invernizzi, Milan, Italy; c Istituto di Ricovero e Cura a Carattere Scientifico Fondazione Santa Lucia, Rome, Italy

**Keywords:** Epigenetics, Emery Dreifuss Muscular Dystrophy, Lamin A/Cmechanotrasduction, nuclear architecture, transcription

## Abstract

The alteration of the several roles that Lamin A/C plays in the mammalian cell leads to a broad spectrum of pathologies that – all together – are named laminopathies. Among those, the Emery Dreifuss Muscular Dystrophy (EDMD) is of particular interest as, despite the several known mutations of Lamin A/C, the genotype–phenotype correlation still remains poorly understood; this suggests that the epigenetic background of patients might play an important role during the time course of the disease. Historically, both a mechanical role of Lamin A/C and a regulative one have been suggested as the driving force of laminopathies; however, those two hypotheses are not mutually exclusive. Recent scientific evidence shows that Lamin A/C sustains the correct gene expression at the epigenetic level thanks to the Lamina Associated Domains (LADs) reorganization and the crosstalk with the Polycomb Group of Proteins (PcG). Furthermore, the PcG-dependent histone mark H3K27me3 increases under mechanical stress, finally pointing out the link between the mechano-properties of the nuclear lamina and epigenetics. Here, we summarize the emerging mechanisms that could explain the high variability seen in Emery Dreifuss muscular dystrophy.

## Introduction

Complex organisms are formed of several specialized cell types, each having characteristic features. Their phenotypic diversity is due to the cell-specific expression pattern of gene subsets. At the single gene level, the transcriptional state is determined by epigenetic mechanisms of regulation that establish distinct layers of structural organization, including covalent modification of DNA and histones, packaging of DNA around nucleosomes, higher order chromatin interactions and nuclear positioning [1]. The ensemble of epigenetic mechanisms determines the epigenome complexity, which establishes and maintains the cell identity in time and space without altering the DNA sequence. To achieve higher order configurations and/or to maintain the steady state of chromatin conformation, epigenetic factors cooperate with nuclear structures, such as nuclear pores and the nuclear lamina [2]. In fact, recent evidence has described how, in several human pathologies, alterations of nuclear components can influence genome conformation with drastic consequences for gene regulation [2]. For this reason, in these years, the study of the crosstalk between the epigenome and the nuclear architecture has attracted considerable interest. In this review, we will summarize the recent findings concerning genetic and epigenetic dysfunctions in Emery Dreifuss Muscular Dystrophy (EDMD), a human disease caused by mutations in the components of the nuclear lamina.

## The nuclear lamina

The nuclear lamina (NL) is a branched protein meshwork beneath the inner nuclear membrane (INM). The principal components of the NL are lamins, intermediate filaments (IF) proteins of the V type. Lamin proteins exhibit a tripartite structure, constituted of an α-helical central rod domain flanked by N-terminal and C-terminal globular domains [3–5]. In vertebrates, lamins are divided into B-type, encoded by *LMNB1* and *LMNB2* genes, and A-type, encoded by *LMNA* gene [6]. The alternative splicing of *LMNA*-derived transcripts generates two main proteins: Lamin A and Lamin C [7,8]. Lamin A is initially synthesized as a longer precursor, the prelamin A, which undergoes a series of post-translational modifications inside the nucleus: the CaaX motif at its C-terminus is farnesylated, partially cleaved and then carboxymethylated. Finally, an endoproteolytic cleavage produces the mature Lamin A protein [9]. Mature Lamin A can dimerize with a parallel and in-register homolog via a coiled-coil structure which involves the four α-helical segments of their central rod domains [10]. Anti-parallel homodimers then assemble in a head-to-tail fashion forming protofilaments, whose weaker lateral half-staggered interactions generate IF-like structures of heterogeneous diameter [10,11]. Transmission electron microscopy, in 1986, afforded the first structural view of nuclear lamins: nuclear envelopes isolated from Xenopus laevis oocytes revealed IF-like filaments with an approximate diameter of 10-nm forming a net, with the two orthogonal sets of filaments having a crossover spacing of about 52 nm [12]. These observations were later confirmed by using cryoelectron and field emission scanning microscopies [13,14]. More recently, Turgay and colleagues used cryo-electron tomography (cryo-ET) in vimentin-null mouse embryonic fibroblasts to show that A- and B-type lamins localized under the INM exhibit a peculiar structure, assembling into tetrameric filaments of 3.5 nm thickness [15]. This structure is different from other canonical cytoskeletal elements such as microtubules, vimentins and actin, that form thicker filaments. Three-dimensional structured illumination microscopy (3D-SIM) was also used to visualize lamin organization [16] and highlighted a type-specific distribution of lamins, possibly suggesting that different lamin isoforms have distinct roles in maintaining the organization of the nuclear lamina.  In line with these findings, previous works showed that lamin proteins are not exclusively present at the nuclear periphery, but also localize in the nucleoplasm [17–19]. Unfortunately, cryo-electron tomography studies were restricted to peripheral lamins [15]. Thus, further studies, combining new imaging technologies, will hopefully elucidate the real structure of lamins in the nucleoplasm and will define in detail how the network of different lamin isoforms organized in distinct nuclear spaces.

### The nuclear lamina as a chromatin organizer

Due to its structure and positioning underneath the INM, the nuclear lamina was initially thought as a static skeletal element involved in the organization of the nuclear envelope [20,21] and in the definition of higher order chromatin domains in interphase chromosomes [21–24]. Then came their suggested role as an assembly platform that could connect and coordinate the complex molecular machineries involved in a wide range of functions [25]. This hypothesis is supported by a burst of experimental evidence that linked Lamin A/C to active cell processes, including cell migration [26], signal transduction [27–29], RNA PolII-dependent transcription [30], DNA replication [31,32], cell cycle [33,34] and cancer growth [35-37]. Lamin proteins are also involved in the epigenetic regulation of chromatin [38–41], as evidenced by the fact that nuclear periphery is widely recognized as a repressive environment [42,43] and lamins directly interact with the genome at specific DNA sequences called Lamina Associated Domains (LADs) [44]. These domains, of a variable length from 0.1 to 10 Mb, create an environment that maintains genes repressed [45,46] and marked by H3K9me2 and H3K9me3 histone modifications [47–49]. The borders of LADs include gene promoters that often contain H3K27me3 histone modification, suggesting a peculiar lamin dependent chromatin organization where the transcriptional start sites of genes located in the facultative heterochromatin are adjacent to constitutive heterochromatin [50]. Some LADs, called constitutive LADs (cLADs), are associated with the lamina across cell types and conserved between species, suggesting a role in the organization of chromosomal architecture [44–46]. Other LADs, the facultative LADs (fLADS), are cell-specific, pointing out a possible role in cell identity and differentiation. Repositioning of silent genes to the nuclear periphery [51] and the crosstalk with developmentally regulated transcriptional factors in the nucleoplasm [52,53] both indicate that the nuclear lamina plays a role in transcriptional regulation and cell identity specification. For instance, during the differentiation of adipose stem cells (ASCs) into adipocytes, genes that regulate the adipogenesis are released from the lamina while genes that maintain the cells undifferentiated are retained near to it, in a repressive environment [49]. Similarly, during the differentiation of embryonic stem cells (ESCs) into neural precursor cells (NPCs), “stemness genes” such as *Nanog*, *Klf4* and *Oct4*, which are progressively repressed, exhibit significantly increased interactions with the nuclear lamina in NPCs compared to ESCs [54]. This ability of lamins to influence the localization of genes suggests a role for nucleoplasmic Lamin A/C in pulling genomic regions toward the nuclear center [49]. It is also possible that the interaction between nucleoplasmic Lamin A/C and LADs are dynamic and temporary acting in “intermittent molecular contact” as already shown for the peripheral Lamin A/C fraction [47].

## Emery dreifuss muscular dystrophy (EDMD)

Since Lamin A/C covers such a variety of roles, it is not surprising that mutations in the *LMNA* gene cause a wide set of pathologies, grouped all together under the term of Laminopathies [55]. Lamin A-dependent diseases are tissue-specific and can affect the nervous system [56], the striated muscle [57–59], the cardiac muscle [60,61] or the adipose tissue [62,63]. Other laminopathies lead to premature aging diseases as in the case of Hutchinson-Gilford Progeria Syndrome (HGPS) [64] and atypical Werner syndrome (AWS) [65]. In minor part, also B-type Lamin takes part to Laminopathies giving rise to neuropathy and lipodystrophy [66].

Lamin A/C dependent Laminopathies affecting the striated muscles have been described as a continuous spectrum of successive phenotypes. A strong correlation between age of onset and the specific mutation, but also the severity of the phenotype has been described [67]. In general, the early prenatal onset is associated with lethal fetal akinesia, late prenatal onset with severe lamin-related congenital muscular dystrophy (L-CMD), onset before 1 year with dropped head L-CMD, onset in childhood or young adulthood with classic EDMD, later onset with LGMD1B, and finally, the end of the spectrum where no skeletal muscle involvement is noted [68,69].

EDMD is thought to be the third most common dystrophy, following Duchenne and Becker muscular dystrophies. The prevalence of EDMD has been estimated at 0.13:100,000 – 0.2:100,000 [67]. The vast majority of EDMD-causing variants present an autosomal dominant inheritance: they consist of single nucleotide mutations, short insertions or deletions in one of the two LMNA alleles (AD-EDMD). Some of them affect residues in domains required for intra- or intermolecular interactions; others introduce a premature stop codon yielding a truncated, dysfunctional protein [68]. A smaller proportion of patients have an X-linked recessive form (XL-EDMD), associated with mutations in the *EMD* gene, coding for Emerin, an integral protein of the inner nuclear membrane located on the X chromosome [70]. Among the mutations reported, most are predicted to cause loss of protein expression, while few of them are missense. At present, only five patients affected by autosomal recessive form (AR-EDMD) have been identified [59,71]. The first patient showed difficulties when he started to walk due to severe muscular dystrophy and joint contractures; he stopped walking by the age of 5 years but showed no cardiac involvement [59]. The other four individuals have a homozygous c.674G>A LMNA pathogenic variant and belong to the same family, in which one sibling was accidentally diagnosed with AR-EDMD [71]. These patients have a severe muscular dystrophy involving proximal muscles around the hips and shoulders (limb-girdle dystrophy); two of them – 25 and 35 years old – have joint contractures causing loss of ambulation; premature atrial and ventricular contractions and conduction defects have been diagnosed.

EDMD usually manifests itself in patients between mid-childhood and the second decade of life with slowly progressive muscular weakness, joint contractures, and cardiac disease [70]. AD-EDMD and XL-EDMD are characterized by three diagnostic principles: i) early contractures affecting selectively the Achilles tendons, the elbow flexors and neck extensors; ii) a limitation of extension of the whole spine, due to the progressive development of spinal cervicodorsal; iii) lumbar contractures [68]. In XL-EDMD, joint contractures are usually the first sign, whereas, in AD-EDMD, they may appear after the onset of muscle weakness [68]. Variable slowly progressive muscle wasting and weakness occur, starting in humeroperoneal/scapulo-peroneal muscles and then extending to the lower legs. Loss of ambulation can occur in AD-EDMD but is rare in XL-EDMD [68]. Cardiomyopathy at later stages of the disease is associated with conduction abnormalities. In AD-EDMD, the risk for ventricular tachyarrhythmia and dilated cardiomyopathy manifested by left ventricular dilation and dysfunction is higher than in XL-EDMD [72]. The cardiac conduction system can be affected at all levels and can manifest itself as sick sinus syndrome, atrioventricular block or bundle branch blocks. This leads to the necessity for implantation of a pacemaker; furthermore, the presence of arrhythmias usually precedes the chamber enlargement [73]. The course of the cardiomyopathy is aggressive and leads to premature death. By an age of 60 years, 55% of *LMNA* mutation carriers die of cardiovascular death or receive a heart transplant, compared with 11% of patients with idiopathic cardiomyopathy without *LMNA* mutation [74].

Women heterozygous for mutations in EMD gene usually do not show any major clinical symptoms and are therefore named ‘healthy carriers’ of XL-EDMD. However, skin sections from healthy carriers showed a mosaic pattern of emerin expression in immunofluorescence assays and western blot analysis on their blood cells revealed reduced levels of emerin protein [75]. Drastic reduction in emerin levels was initially associated with some cases of cardiac involvement among healthy carriers [75-78]. In fact, together with the cardiac phenotype, a great reduction in Emerin levels with respect to non-symptomatic females was observed. Methylation-sensitive restriction enzyme assay suggested that the clinical manifestation was due to uneven X-inactivation [75]. However, more recently, Meinke and colleagues described a mild muscular and cardiac phenotype in healthy carriers not associated with X-inactivation defects [79]. They excluded a dominant-negative effect of the mutant truncated peptide since its expression was undetectable in western blot of patient myoblasts. Furthermore, the patient's grandmother, a carrier in her own right, was unaffected. A second, modifying mutation was therefore hypothesized but, unfortunately, not identified. The concrete opportunity to analyze symptomatic and asymptomatic healthy carriers of EDMD could be a significant advance in the field and could be used to discover secondary mechanisms and/or mutations contributing to the disease phenotype. Moreover, despite the high number of identified pathological mutations, in more than 60% of EDMD cases, no genetic mutations were detected in *EMD* or *LMNA* genes [70], suggesting the presence of other genes involved in EDMD dystrophy. We predict that in the coming years the number of patients diagnosed with AR-EDMD will increase, thanks to the increasing accessibility of next-generation sequencing in clinical medicine.

### EDMD mouse models

The first Lamin A/C knockout mouse (*Lmna* −/−) was generated by the group of Brian Burke in 1999 [80]; the region spanning from exon 8 to part of exon 11 was replaced by a Pgk-neomycin resistance cassette (Table 1). Though homozygous mice show no difference in phenotype compared to the wild type ones at birth, their growth rate decreases within 2–3 weeks and ceases by around 4 weeks. By then, mutant mice display an abnormal gait with a stiff walking posture; scoliosis/kyphosis are documented. By the 8th week, all homozygotes mice die. Although the rapid onset dystrophy is in contrast with lamin dependent dystrophy in humans, this being mainly autosomal dominant and having a slow progression, homozygous mice show several features of the human Emery Dreifuss muscular dystrophy.  Microscopical analysis shows that the paravertebral muscles and some belonging to the hindlimb (more specifically, rectus femoris and semimembranosus) are dystrophic; the fibers proximal to the bone are the most severely impaired [80]. Muscles of the head, tongue, and diaphragm are spared by the dystrophy. In the heart, the ventricular muscle is that most affected, though the involvement of myocytes is variable [81]. In 2012, Jahn and colleagues [82] discovered the presence of a lower amount of a truncated Lamin A in *Lmna* −/− mice. The strain, renamed *Lmna* Δ8-11, cannot be thus considered “null”. This C-terminally truncated Lamin A, although produced in very low amounts, could either be functionally hypoactive and able to carry out only certain functions but fail to complete others (loss of function model) or, alternatively, it could act as a toxic molecule with a dominant-negative effect (gain-of-function model).

**Table 1.** Mouse models of Emery Dreifuss Muscular Dystrophy.

Lmna mouse	Phenotype	Comment
*Lmna* Δ8-11	Homozygous mice show an abnormal gait with a stiff walking posture by the 4^th^ week. Scoliosis/kyphosis are documented.	A truncated isoform of Lmna (Δ8-11) is present; strain cannot be considered “null”.
*Lmna* GT	Homozygous mice hunched posture and abnormal gait, characterized by splayed hind legs from day postnatal day 13.	No truncated isoform is present; can be thus considered *Lmna* null.
*Lmna* H222P	At adulthood, male homozygous mice display reduced locomotion activity with abnormal stiff walking posture.	Heterozygous *Lmna* H222P causes autosomal dominant EDMD in humans
*Lmna* ΔK32	Homozygous mice show a delay in striated muscle maturation, reduced fat tissue and hypoglycemia leading to premature death (by the 3rd week).	The loss of lysine 32 in the N-terminal domain of *LMNA* leads to a severe congenital muscular dystrophy (CMD).

To overcome artefact effects due to this truncated Lamin A form, another mouse strain, the *Lmna* GT −/−, was generated in 2011 [83] by interruption of the endogenous Lamin A/C locus with a promoter-trap construct, which introduces an in-frame *Lmna*-βgeo fusion allele into *Lmna* intron 2 followed by β-galactosidase-neomycin cDNA (Table 1). Interestingly, by following β-galactosidase staining authors detected the activity of *Lmna* promoter in the heart, liver and embryonic somites at E11, thus dispelling the myth that Lamin A is expressed only after birth. *Lmna* GT −/− mice display a phenotype similar to the previous *Lmna* Δ8–11: hunched posture and abnormal gait, characterized by splayed hind legs from day P13 onwards. At the histological level, post-natal hypertrophy of cardiac myocytes and hypotrophy of the quadriceps are present. *Lmna* GT −/− mice die at 16–18 days after birth, much earlier than *Lmna* Δ8–11 mice, and representing the most severe loss-of-function mutants for A-type lamins to date. Consistent with findings obtained in these mouse models, homozygous *LMNA* nonsense mutation has been detected in a newborn patient leading to the complete absence of Lamin A/C; the child died at birth due to respiratory insufficiency and severe generalized muscular dystrophy [84].

Another mouse model for EDMD was created in 2005 by Arimura and colleagues: the *Lmna* H222P/H222P knock-in mouse [85] (Table 1). H222P is the first missense mutation discovered to be associated to EDMD. A transversion (CAT>CCT) in exon 4 of *Lmna* leads to a change from histidine to proline at codon 222 (c.665A>C, p.H222P). The mutant mice do not show any developmental anomaly and reach sexual maturity, but at adulthood, starting at approximately 16 weeks, male homozygous mice develop muscular dystrophy with abnormal stiff walking posture and all of them die by the 9th month. Dilative cardiomyopathy and hypokinesia with conduction defects are present; the cardiac involvement in female homozygous mice arises later compared to the male counterpart. A marked increase in fibrosis occurs in the heart of homozygous mice; degeneration and necrosis affect cardiac muscle cells in the ventricles. At the skeletal muscle level, variability in fiber size with hypertrophic muscle cells and numerous degenerative fibers can be observed in both diaphragm and soleus of male homozygous mice [68]. However, in contrast with the *Lmna* null mouse, only a moderate phenotype was observed in gastrocnemius, quadriceps, triceps and tibialis anterior as reported by the author (data not shown). For the variable and late onset of muscular dystrophy, this mouse has mainly been used for studies on the heart.

In 2011, Bertrand and colleagues created the *Lmna* ΔK32 knock-in mouse; this strain harbors a severe congenital muscular dystrophy (L-CMD) mutation [86] that leads to the loss of lysine 32 in the N-terminal domain of *LMNA* (Table 1). Though at birth *Lmna* ΔK32/ΔK32 mice do not show any difference from the wild type littermates, they display a severe reduction in the growth curve already at P5; by P15, only half of the homozygous mice survive and by P19 they are all dead. Homozygous mice show severe metabolic defects and a waddling gait with an increasing number of falls, probably due to hindquarter blockade. Microscopically, all muscles analyzed are affected, showing a reduction of fiber size associated with the presence of central myonuclei, as documented by the analysis of the gastrocnemius at P14, and indicating a delay in skeletal muscle maturation [86].

Interestingly while all described mouse models carrying mutations or deletion of Lamin A do not show disease features in heterozygous, some evidence reported progressive electrophysiological cardiac abnormalities commencing around 4 weeks after birth and the emergence of late-onset cardiomyopathy in aged (50 weeks) heterozygous *Lmna* Δ8-11 mice, suggesting a cardiac pathology [87]. Telemetric and in vivo electrophysiological studies in 10-week-old *Lmna* Δ8-11 +/− mice showed AV conduction defects and both atrial and ventricular arrhythmias, analogous to those observed in humans with heterozygous *LMNA* mutations. On the other hand, neonatal heterozygous *Lmna* Δ8-11 mice do not show conduction system defects suggesting that the heart development is not affected [87]. A similar phenotype has been observed in heterozygous *Lmna* ΔK32mice [88]. Haploinsufficiency of Lamin A in cardiomyopathy affected patients has also been described [89,90], suggesting that heterozygous mice can recapitulate some features of the human disease.

Taking into consideration that Lamin A is a key component of the nuclear environment and interacts with many other factors, it is not surprising that knock-out of known Lamin A-interactor in mouse, show muscular phenotypes similarly to lamin mutant mice. The study of these mouse models could shed a light on distinct Lamin A functions and their role in muscular dystrophy pathogenesis and progression. Lap2α is a nucleoplasmic protein implicated in cell cycle regulation through its interaction with A-type lamins and the retinoblastoma protein; a heterozygous mutation in *Lap2α* affecting its Lamin A/C interaction domain has also been linked to a cardiomyopathy [91], symptomatically similar to Lamin A/C-linked ones [92], suggesting a common disease mechanism of these two proteins. *Lap2α* −/− mice are not dystrophic and their heart is not affected histologically [19]. However, Gotic and colleagues showed in primary myoblasts that the loss of Lap2α interferes with the process of the myofiber-type specification, though muscle morphology, function, and regeneration are not compromised *in vivo* [93]. More specifically, slow muscles in *Lap2α* −/− exhibit a shift toward the fast fiber phenotype. Interestingly, fast fiber shifts were also observed in skeletal muscles of patients with chronic heart failure [94]. Atrophy of slow fibers occurs also in *Lmna* H222P/H222P skeletal muscles and in some human striated muscle laminopathies where anomalies in heart structure and function are also present [95,96]. The predominantly oxidative metabolism of both slow skeletal and cardiac muscle ^94^ affected in these conditions suggests a role of lamins and their interaction partners in the regulation of oxidative metabolism of myofibers and postnatal muscle remodeling.

### Molecular mechanisms underlying EDMD

In laminopathies, the symptomatology differs considerably among patients, even in the same family, so suggesting that the individual epigenetic background may play a major role in the disease development [97,98]. In line with this hypothesis, aberrations in the transcriptional control have been described in several laminopathies [99]. Most lipodystrophies, for example, share impaired interaction of Lamin A with SREBP1, an important transcription factor for lipid homeostasis [100]. On the other hand, lamin dependent muscular disorders show disruption of the transcriptional programs supported by the MAPK, pRb, MyoD, Wnt-β catenin and TGFβ altered pathways [68, 101–103]. Muscle regeneration and growth benefit from a small population of heterogeneous stem cells named Satellite Cells (SCs) that in normal condition rest in a quiescent state under the basal lamina of the adult muscle fibers [104]. SCs express α7-integrin surface marker [105,106] and Pax7 (Paired Box Protein Pax-7) intranuclear transcription factor [107–109]. In response to various physiological or pathological stimuli, quiescent SCs are activated by expressing MyoD (myogenic determination protein) and can adopt divergent fate decisions that result in differentiation or self-renewal. The differentiation program ensures muscle regeneration, growth and myofibers turnover while the self-renewal preserves the muscle stem cell niche from the exhaustion [110]. Epigenetic mechanisms sustain and influence both SCs fate choices being at the intersection of cell specification and identity. Key regulators of muscle differentiation are the Polycomb Group of proteins (PcG). These proteins are evolutionary conserved epigenetic repressors that act at different levels of epigenome complexity from histone modification to chromatin remodeling, ensuring the establishment and the maintenance of cell identity [111].  PcG proteins aggregate into different complexes working together or separately on chromatin targets [112]. Polycomb Repressive Complex 1 (PRC1) deposits the repressive histone mark H2aK119ub through the catalytic subunit Ring1a/b and Polycomb Repressive Complex 2 (PRC2) is responsible for H3K27me3 through the catalytic activity of Ezh2. At the microscopic level, PcG proteins form aggregates (PcG bodies) [113,114], an intranuclear architecture necessary for mediating the chromatin long-range interactions and the clustering of their targets [52,113,115]. In muscle, PcG proteins finely regulate and coordinate muscle genes expression ensuring cell identity, differentiation and self-renewal [116–118]. In particular, PcG proteins maintain repression at myogenic markers in undifferentiated cells while, at the onset of differentiation, they relocate on stemness genes [116,119]. In muscle cells, chromatin relaxation and accessibility for transcription factors are also regulated by histone acetyltransferases (HATs) and deacetylases (HDACs) that respectively stimulate and repress gene transcription [120]. During muscle differentiation, HATs and HDACs act analogously to PcG, relocating to the specific targets in a time-specific manner [116,121–123]. In particular, during differentiation, the HAT p300 acetylates the regulatory element of MyoD gene, stimulating its expression [124], essential for myogenesis [125]. Interestingly, besides alternative PRC complexes binding to specific targets, plasticity in PRC complex composition also ensures proper maintenance of SCs transcriptional program and the response to environmental stress [117,118,126–128]. For instance, during the terminal differentiation, the proper timing for *MyoG* transcription is regulated by the Ezh1, the alternative catalytic subunit of PRC2, that replaces Ezh2 on *MyoG* promoter [129]. Interestingly Ezh1 is also directly involved in the stress response in muscle: Bodega et colleagues described a novel isoform of Ezh1, the Ezh1β, that acts in the cytoplasm of post mitotic skeletal muscle cells as a stress-sensor and controls nuclear PRC2 activity [126]. In response to atrophic oxidative stress, cytoplasmic Ezh1β releases the PRC2 subunit Eed from cytoplasm allowing its correct nuclear assembly with Suz12 and the canonical Ezh1 form, the Ezh1α, on myogenic promoters. This dynamic is necessary to prime an epigenetic response and to protect cell integrity.

Since Lamin A/C can directly bind DNA, shuffling the chromatin from repressive to permissive transcriptional environment and vice versa, it has been proposed that in laminopathies this epigenetic mechanism could be affected. Several studies have reinforced this hypothesis: i) an aberrant heterochromatin localization at the nuclear envelope and loss of the myogenic program have been found in murine *Lmna*-null cells [41]; (ii) expression of the human EDMD LMNA in Caenorhabditis Elegans impairs tissue-specific reorganization of heterochromatin, with abnormal retention of a muscle-specific, transcriptionally silent gene at the nuclear periphery [130]; iii) nuclear positioning of the PcG regulated FSHD (Facioscapulohumeral muscular dystrophy) locus, responsible for an autosomal dominant neuromuscular disorder, is altered in human Lamin A/C null cells [131]; iv) the R482W mutation on Lamin A causing familial partial Dunnigan lipodystrophy (FPLD2) prevents Lamin A binding at MIR335 locus and mediates enhancers looping; this determines the aberrant transcription of the anti-adipogenic miR-335 with subsequent inhibition of adipogenic differentiation [132]; v) Robson and colleagues showed that, during myogenesis, nuclear envelope transmembrane proteins (NETs) redirect the correct positioning of myogenic genes; in NET knockdowns such repositioning is affected and myotube formation is blocked [133].

More recently, Perovanovic and colleagues, with a genome-wide approach based on DamID-seq technology [134], investigated the mechanisms affecting cell differentiation in laminopathies [135]. They found that R453W and R482W mutations, responsible for Emery Dreifuss Muscular Dystrophy (EDMD) and familial partial lipodystrophy (FPLD) respectively, disrupted the appropriate formation of Lamin A–associated heterochromatin domains in an allele-specific manner. Perturbations of the epigenomic transitions determine some recurrent aberrations at the transcriptional level, including lack of pluripotency and induction of myogenic loci, suggesting that in EDMD the altered formation of lamina-associated domains affects the epigenetic programming. Other studies investigated the direct crosstalk between Lamin and epigenetic factors. Super-resolution microscopy analysis revealed an interrelated distribution between PcG proteins and nuclear Lamin A/C [52]. Their relative intranuclear localization is not random; rather Lamin A/C surrounds the PcG bodies ensuring their correct organization and positioning on target genes mirroring the correct gene expression. Knock down of Lamin A/C in muscular cells determines PcG bodies disassembly and an anticipated muscle differentiation [52,53]. Further studies in pathological models will hopefully elucidate how the mechanisms described here are involved in laminopathies and which is their interplay.

### Epigenetics and mechanotransduction

At the cellular level, Lamin A/C is one of the most important components of mechanotransduction, the mechanism by which external mechanical stimuli are converted into biochemical intracellular signals [136,137]. Studies on isolated nuclei showed that exposed to shear stress, the Ig fold domain of Lamin A is able to partially unfold, leading to stretching of the molecule [138,139]. Moreover, when the extracellular matrix increases its stiffness, Lamin A levels increase and the protein undergoes dephosphorylation of the S22 residue. The nucleoplasmic portion relocates to the nuclear lamina, providing increased nuclear stiffness and mechanical support [136,139–141]. Considering these observations, for several years, scientists were convinced that lamin-dependent diseases were a consequence of aberrant mechanotransduction due to a higher sensitivity of the mutant to the mechanical stress [142,143]. This hypothesis was supported by evidence that, in the context of constant mechanical stress, mutant lamin protofilament could be more prone to dissociation [80,144–146], determining muscular specific nuclear rupture, cell death and tissue deterioration [147,148]. In line with these observations, microarray analysis conducted on the hearts of *Lmna* H222P/H222P mice showed that the extracellular signal-regulated kinase 1/2 (ERK1/2) and the Jun N-terminal kinase (JNK) signaling pathways, both being branches of the stress-related, mitogen-activated protein kinase (MAPK) cascade, are abnormally activated [149]. Furthermore, other signaling cascades, AKT/mTOR [150], TGF-β, CTGF [151] and p38α [152], are involved in the pathogenesis of the EDMD cardiomyopathy. On the other hand, the mechanotransduction process implies the translation of external signals into transcriptional changes. Lamin A, using its direct association with DNA at Lamina-Associated Domains (LADs), could orchestrate the genome reprogramming, directly linking mechanotransduction to chromatin. This would explain the emerging findings that attribute some features of laminopathies to genome misregulation, from failure in stem cell maintenance to impaired differentiation [45,153–156]. Interestingly, PcG proteins are also involved in the mechano-signal transduction mediated by Lamin A [157]. As described above, mechanical stress induces a reshaping of the nuclear lamina composition, this triggering a decrease in H3K9me2,3 and an accumulation of H3K27me3 with inhibition of RNA Polymerase II [157]. This observation paves the way for a new hypothesis: in lamin mutant cells, a defective Lamin A/PcG crosstalk and consequent programming dysfunctions could finally account for the higher sensitivity of these cells to mechanical stress. It is therefore tempting to speculate that, in Laminopathies, the uncoupled mechanotransduction axis and alterations at the chromatin level may be closely connected to common Lamin A dysfunctions.

### Epigenetic therapies for muscle disorders

Epigenetic treatments for muscle disease are attractive opportunities to tackle these pathologies; indeed, some clinical trials are already ongoing with promising results. In particular, in Duchenne Muscular Dystrophy, an X-linked pathology related to mutation of Dystrophin (DMD), HDAC inhibitors have revealed beneficial effects [158]. In particular, the HDAC inhibitor (HDACi) Givinostat has been tested on the mouse model of DMD showing an improvement in muscle homeostasis and regeneration [159]. Mechanistically, HDACi directly prevents the deacetylation and inactivation of MyoD protein, thus enhancing muscle regeneration in DMD [160–162]. At the DNA level, HDACi prevents histone deacetylation, thus maintaining Myod1 and Mef2 (Myocyte enhancer factor 2) loci transcriptionally active. This will sustain the transcriptional cascade leading to muscle differentiation [124,163,164]. Moreover, HDACi favors the expression of Follistatin, an antagonist of the muscle differentiation and growth inhibitor, Myostatin [165-167]. These encouraging results fostered the beginning of a clinical trial that is currently entering phase II [168].

Signaling pathways are known to impact on chromatin conformation by targeting chromatin modifying proteins [169]. Among the players involved in the EDMD cardiomyopathy, p38α is known to phosphorylate Ezh2 in satellite stem cells, thus promoting the repression of Pax7 during myogenesis [119]. On the other hand, the PI3-AKT pathway both reduces the affinity of the PRC2 for histone H3 and increases the H3K27-specific acetyl-transferase activity of P300, thus contributing to the switch from a repressive to an activated state of lysine K27 residue of histone H3 [169]. Several compounds involved in the aberrant signaling have been tested. On the basis of scientific findings on the *Lmna* H222P/H222P mice [85] Muchir and colleagues treated H222P/H222P mice with small molecules inhibiting the phosphorylation of ERK and JNK (PD98059 and SP600125) before and after the onset of the cardiomyopathy; the same authors documented a delay in left ventricular dilation, an improvement in the ejection fraction (EF) and a decrease in myocardial fibrosis [170–172]. Unfortunately, those molecules are not suitable for use in patients due to problems with bioavailability and toxicity [171]. Subsequently, Selumetinib was used to inhibit ERK1/2 signaling as it has been safely administered to human subjects in clinical trials for cancer [173]; its use led to cardiac fractional shortening, improvement of survival and prolonged overall survival in Lmna H222P mice. In 2014, Selumetinib was used in conjunction with Benazepril, an angiotensin II converting enzyme (ACE) inhibitor that constitutes standard medical therapy for patients with heart failure. When combinatory treatment started at 16 weeks of age, after the onset of left ventricular dysfunction, a statistically significant increase in left ventricular fractional shortening at 20 weeks of age was documented in H222P/H222P mice [174]. To our knowledge, no clinical trial to target *LMNA* cardiomyopathy with Selumetinib has been conducted to date.

The hyperactivation of a third branch of the MAP kinase cascade, p38α signaling, was identified in the hearts of *Lmna* H222P/H222P mice [152]. These mice were treated with a p38α inhibitor, named ARRY-371797. In parallel, a placebo was administered to another group of H222P/H222P mice as negative control. Scientists found that ARRY-371797 treatment prevented left ventricular dilatation and deterioration of fractional shortening. A phase 2 clinical trial with ARRY-371797 started in February 2014 (NCT02057341) for patients with symptomatic genetic dilated cardiomyopathy due to Lamin A/C gene mutation (https://clinicaltrials.gov/ct2/show/NCT02057341?term = NCT02057341&recrs = abeh&rank = 1). The preliminary study is complete and a rollover one (NCT02351856) will be conducted until December 2018 (https://clinicaltrials.gov/ct2/show/NCT02351856?recrs = abeh&cond = ARRY-371797&rank = 8?). In 2017, a novel macrocyclic MEK1/2 inhibitor with improved pharmacological profile was synthesized and proved to be effective in ameliorating the outcome of the cardiomyopathy [175].

The observation that AKT-mammalian target of rapamycin pathway is hyperactivated in the *Lmna*-mutation-caused cardiomyopathy has paved the way for the *in vivo* administration of the rapamycin-analog temsirolimus [150]; the inhibition of mTORC1 inhibition reactivates autophagy and prevents cardiac damage in *Lmna* H222P/H222P mice. These data are reinforced by those of Ramos and colleagues on rapamycin treatment of *Lmna* −/− mice [176]. Eventually, the activation of Wnt/β-catenin activity with BIO (6-bromoindirubin-3'-oxime), a reversible and ATP-competitive inhibitor for GSK-3α/β, improved cardiac contractility and ameliorated intraventricular conduction defects in *Lmna* H222P/H222P mice [102]. New epigenetic studies are required in the mouse models of laminopathies, more specifically before and after treatment with the above-cited small molecules, in order to further elucidate the mechanisms downstream the alteration of the signaling pathways. This could allow both a “surgical” intervention on the single mechanism and combinatory approaches acting on several levels from signaling to gene regulation.

## Conclusion

**Figure 1.**
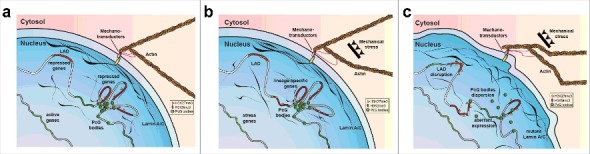
Schematic representation of Lamin dependent mechanotransduction. Nuclear lamina works in couple with the mechanotransductor machinery to convert mechanical stimuli into epigenetic changes including: Histone modification, LAD higher order structures and PcG organization (a, b) In EDMD, the compromised mechano-properties of aberrant form of Lamin A/C could increase the sensitivity of the nuclei to mechanical stress resulting in: loss of LAD conformation, PcG bodies dispersion and aberrant gene expression.

Decades of study have unveiled the multiple roles of Lamin A/C, suggesting how aberrant forms of this protein can cause tissue-specific laminopathies. Emerging findings describe the key role of Lamin A/C in transcriptional control, so opening the “Pandora's box” of the multiple epigenetic mechanisms involved in laminopathies. In parallel, basic research studies elucidate the epigenetic involvement in mechanotransduction, linking another Lamin A function with transcriptional control. Indeed, under mechanical stress, the rearrangement of nuclear lamina components mirrors the epigenetic accumulation of PcG mark H3K27me3 [157]. All these findings progressively converge into a unique model by which the uncoupling of the mechanical-properties/epigenetic-factors/gene-regulation axis could drive the tissue-specific pathology (Figure 1). Medical treatment aimed at reversing epigenetic aberrancy is a fascinating path that will hopefully lead to new cures for a whole variety of diseases. However, considering that, in an adult organism, epigenetic regulators are strictly required for the maintenance of the adult tissue self-renewal [117], focused disease-specific studies describing how these regulators participate in the aberrant phenotype could open the way for compounds that specifically inhibit disease-related activity without affecting the other functions necessary for tissue homeostasis.
